# Qualitative and Quantitative Analysis of Phenolic Compounds in Spray-Dried Olive Mill Wastewater

**DOI:** 10.3389/fnut.2021.782693

**Published:** 2022-01-05

**Authors:** Cinzia Benincasa, Massimiliano Pellegrino, Elvira Romano, Salvatore Claps, Carmelo Fallara, Enzo Perri

**Affiliations:** ^1^CREA Research Centre for Olive, Fruit and Citrus Crops, Rende, Italy; ^2^CREA Research Centre for Animal Production and Aquaculture, Bella Muro, Italy; ^3^Olearia S.O.D., Mosciano Sant'Angelo, Italy

**Keywords:** olive mill wastewater, phenolic compounds, spray drier, functional foods, zootechnical products, mass spectrometry

## Abstract

The processing of olives for oil production generates the most abundant agro-industrial by-products in the Mediterranean area. The three-phase olive oil extraction process requires the addition of a large amount of water to the system, which is difficult to dispose of for its load of toxic pollutants. On the other hand, olive mill wastewater is a rich source of bioactive substances with various biological properties that can be used as ingredients in the food industry for obtaining functional and nutraceutical foods as well as in the pharmaceutical industry. In this study, we present the results relative to the phenolic compounds detected in dried olive mill wastewaters obtained using a spray dryer. Qualitative and quantitative analyses were obtained by high-pressure liquid chromatography–tandem mass spectrometry (HPLC–MS/MS). In particular, the compounds here discussed are: apigenin (9.55 mg/kg dry weight), caffeic acid (2.89 mg/kg dry weight), catecol (6.12 mg/kg dry weight), *p*-cumaric acid (5.01 mg/kg dry weight), diosmetin (3.58 mg/kg dry weight), hydroxytyrosol (1.481 mg/kg dry weight), hydroxytyrosyl oleate (564 mg/kg dry weight), luteolin (62.38 mg/kg dry weight), luteolin-7-*O*-glucoside (88.55 mg/kg dry weight), luteolin-4-*O*-glucoside (11.48 mg/kg dry weight), oleuropein (103 mg/kg dry weight), rutin (48.52 mg/kg dry weight), tyrosol (2043 mg/kg dry weight), vanillin (27.70 mg/kg dry weight), and verbascoside (700 mg/kg dry weight). The results obtained highlighted that the use of dehumidified air as a drying medium, with the addition of maltodextrin, appears to be an effective way to produce a phenol-rich powder to be included in food formulations as well as in pharmaceutical preparations having different biological properties.

## Introduction

The processing of olives for oil production generates the most abundant agro-industrial by-products in the Mediterranean area (1). The production, the type, and the quantity of these by-products depend on the extraction system used for the extraction of the oil, which can be in two- or three-phase mills.

The main features of the three-phase extraction system are the need to add a large amount of hot water into the decanter, up to 40 l for every 100 kg of olives processed, to dilute the olive paste, and the production of a severe quantity of vegetation water, up to 100 l for every 100 kg of olives processed (2, 3).

Due to the nature of its compounds, some agricultural and legislative issues must be taken into consideration when vegetation water, i.e., olive mill wastewater (OMWW), is to be used for irrigation purposes (4). OMWW contains high quantities of sugars, tannins, phenolic compounds, polyalcohols, pectins, and lipids, which is highly acidic and toxic for plants (5–8).

Among these, phenols, produced by the secondary metabolism of plants, represent a heterogeneous group of natural substances. Concerning the chemical diversity that characterizes them, phenols play important roles in nature: they act as a defense from herbivorous animals (they give an unpleasant taste) and from pathogens (phytoalexins); offer mechanical support (lignins) and barrier against microbial invasion; act as attractors for pollinators (anthocyanins); and as growth inhibitors of competing plants (2, 3, 7–12).

Many experimental studies have demonstrated phenols to be excellent antioxidants with different biological and pharmacological activities (13–18). Phenols can protect cells from oxidative stress related to different physiological and pathological processes, such as aging, cancer, cardiovascular diseases, and diabetes (16–20). Moreover, they can keep cholesterol levels under control and exert antibacterial, antipruritic, antiparasitic, and cytotoxic properties.

The health–nutritional effects of phenols have been amply demonstrated and its intake varies enormously in relation to the type, quantity, and quality of the vegetables consumed (21–25). Since there are many benefits that phenols bring, it is worth including them in supplements (26).

However, no specific claims have been approved by the European Food Safety Authority (EFSA) concerning phenol-based products. Currently, the only authorization in force is for olive oils containing at least 5 mg of hydroxytyrosol and its derivatives for every 20 g of olive oil (27). Furthermore, EFSA, after evaluating the relationship between acrylamide in food and the increased risk of cancer for consumers of all age groups (28), has proposed several technological approaches to reduce the amount of acrylamide, α-dicarbonyls, and d-AGE in heat-treated foods. Among them, the addition of phenol-based additives has also been proposed (29, 30).

As the object is of growing attention from nutritionists, researchers, and experts, the recovery of phenols is a very current important topic. A low-cost material rich in phenols is OMWW (31, 32). Due to the hydrophilic nature of olive oil by-products and the polarity of phenols, most of them are solubilized in the olive oil residues rather than in the oil. The phenolic composition of OMWW includes more than 50 compounds (2, 3, 25, 33–37) and its total content varies from 0.5 to 2.4 g/l (38), of which 1.2 g/l of hydroxytyrosol and approximately 0.4 g/l of flavonoids (3). Therefore, a suitable method to extract phenols from OMWW could positively affect the economic status of olive companies and reduce the negative impact of olive by-products on the environment.

The recovery of phenols involves typically a condensing step (i.e., thermal concentration, ultrafiltration, or lyophilization) before performing an extraction with organic solvents (i.e., methanol, ethanol, or hydro-alcoholic solutions).

Green methods have also been investigated (Benincasa et al., 2019). Other practices include the application of resin chromatography, selective concentration by liquid membranes, or supercritical fluid extraction (39, 40).

Technologies based on desolvation mechanisms, such as spray drying and freeze-drying to form fine, dry powders, are largely used in the food sector. When thermosensitive molecules have to be encapsulated, freeze-drying is a more interesting alternative to the more commonly used spray drying. However, although freeze drying has the advantage of obtaining high-quality products with minimal thermal and oxidative degradation, it has been shown that the content of phenolic compounds does not undergo significant changes when using spray drying techniques (41, 42). To verify and determine the phenolic content in OMWW, the latter method being faster and having greater industrial flexibility (43) was exploited. In this study, spray drying methodology has been employed to obtain a powder from OMWW which has been extracted for phenolic evaluation and quantitation. High-pressure liquid chromatography–tandem mass spectrometry (HPLC–MS/MS) was the technique employed to determine phenols, such as apigenin, caffeic acid, catecol, *p*-cumaric acid, diosmetin, hydroxytyrosol, hydroxytyrosyl oleate, luteolin, luteolin-7-*O*-glucoside, luteolin-4-*O*-glucoside, oleuropein, rutin, tyrosol, vanillin, and verbascoside.

## Experiment

### Sampling

Olive mill wastewater was collected during the crop year 2018/2019 from the oil mill “La Molazza” located in the southern province of Italy, Cantinella in Corigliano Calabro. OMWW was obtained from the processing of olives of Carolea and Dolce di Rossano varieties after the centrifugation step using a three-phase mill. OMWW was collected in appropriate containers kept in a refrigerated room at 8°C for no more than 3 days. In this way, it was possible to avoid the development of bad odors, slowing down fermentation and oxidation processes that would compromise the quality and quantity of the phenolic compounds it contains.

Olive mill wastewater was dried using a spray drier by “EVRA S.r.L.” company (Lauria, Italy).

### Dried OMWW Phenols Analysis

The analysis of the phenolic compounds was performed as described in a previous work undertaken by Benincasa and co-workers on olive oil pomace (44). Briefly, 20 ml of a solution of methanol/water (v/v 80:20) was added to 20 g of dried OMWW (DOMWW). To help the migration of phenols in the solution, the extraction was conducted in an ultrasonic bath in the darkness and under shaking for 15 min. Centrifugation at 5,000 rpm/min for 25 min was then performed to allow the separation of the phases and the recovery of the supernatant. The remaining residue was re-extracted as explained above two more times.

The resulting supernatants were then combined and analyzed by HPLC–MS/MS.

### Chemicals

All reagents (*n*-hexane, acetone, ethyl acetate, formic acid, and methanol) of LC/MS grade and standards for phenolic assay [catecol (Cat), caffeic acid (Caf), vanillin (Van), *p*-cumaric acid (p-Cum), apigenin (Ap), diosmetin (Dio), hydroxytyrosol (HyTyr), tyrosol (Tyr), oleuropein (Olp), luteolin (Lut), verbascoside (Ver), luteolin-7-*O*-glucoside (Lu7), luteolin-4-*O*-glucoside (Lu4), and rutin (Rut)] were purchased from Sigma–Aldrich (Riedel-de Haën, Laborchemikalien, Seelze, Germany) and Extrasynthese (Nord B.P 62 69726 Genay Cedex, France). Hydroxytyrosyl oleate (HtyOle) was synthesized as reported by Plastina and coworkers (31). Aqueous solutions were prepared using ultrapure water (Millipore, Saint Quentin Yvelines, France).

### Instrumentation

The system used to dry OMWW was a “De Lazzari 5G” spray drier (De Lazzari s.r.l., Busto Arsizio, VA, Italy) set at the following operational parameters: inlet temperature 165°C, outlet temperature <80°C, feeding 4.5 l/h, turbine 2,700 × *g*.

The experimental work was carried out using an HPLC 1200 series instrument (Agilent Technologies, Santa Clara, CA, United States) interfaced to an MSD Sciex Applied Biosystem API 4000 Q-Trap mass spectrometer set in negative multiple reaction monitoring mode (MRM) equipped with an Eclipse XDB-C8-A HPLC column (5 μm particle size, 150 mm length, and 4.6 mm i.d.) for chromatographic separation. The MRM transitions used were: 109 → 91 for Cat; 179 → 135 for Caf; 151 → 136 for Van; 163 → 119 for p-Cum; 269 → 117 for Ap; 299 → 284 for Dio; 153 → 123 for HyTyr; 137 → 137 for Tyr; 539 → 307 and 539 → 275 for Olp; 285 → 133 for Lut; 623 → 161 and 623 → 461 for Ver; 447 → 285 for Lut7 and Lut4; 609 → 301 for Rut; 417 → 281 for HtyOle.

### Calibration Procedure

External calibration curves using a least-squares linear regression analysis were used for the assay of phenols. Standard stock solutions were prepared in methanol and further diluted with water/0.1% formic acid to obtain calibration standards at concentrations in the range between 200 and 3,000 μg/ml. The correlation coefficients of the calibration curve ranged between 0.9997 and 0.9999.

As a direct quantification of oleuropein and ligstroside derivatives was not possible because their standards are not commercially available, their estimate was based on oleuropein glycoside peak areas.

## Results and Discussion

**Figure 1:**
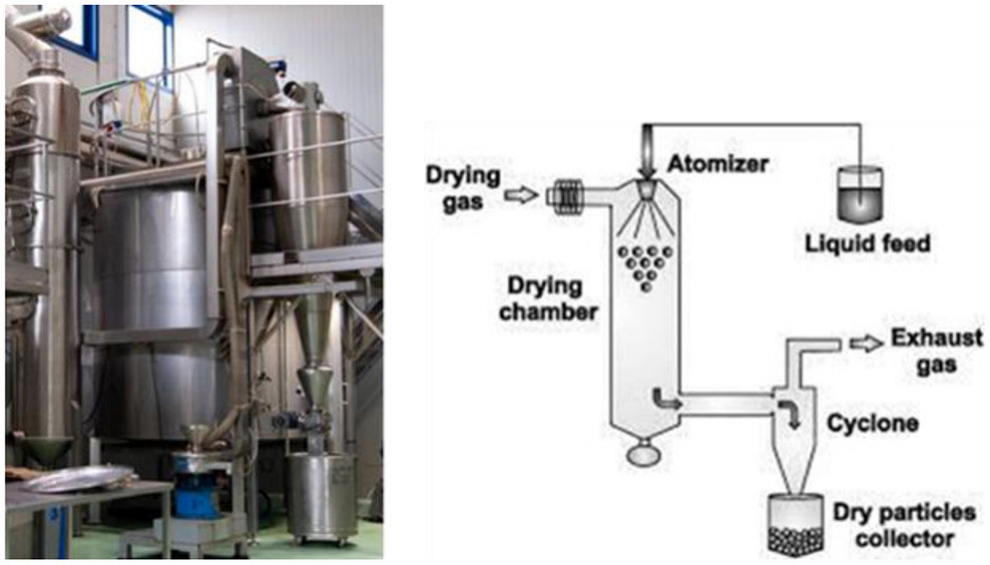
Spray drier system utilized to dry olive mill wastewater (OMWW).

**Figure 2:**
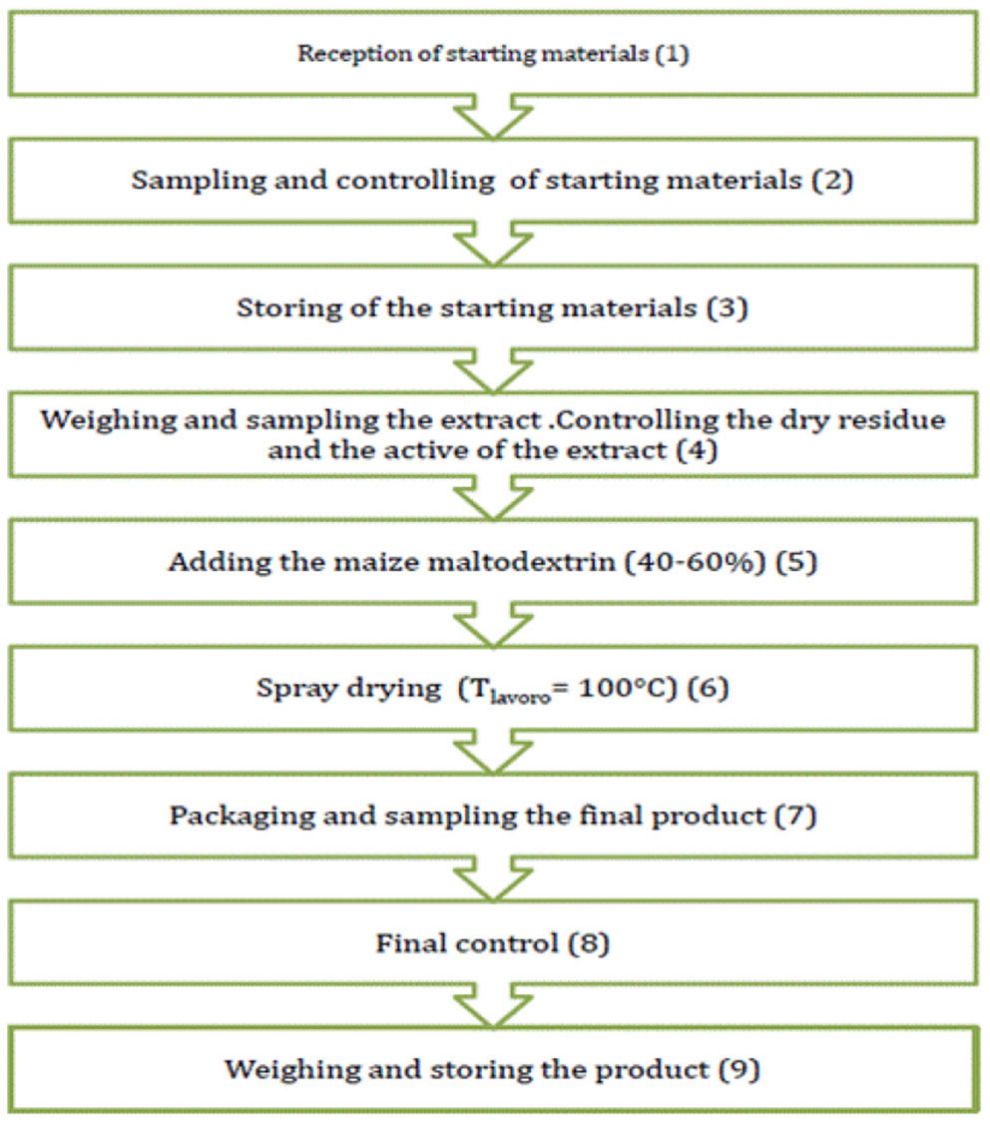
Drying process flow sheet.

**Figure 3:**
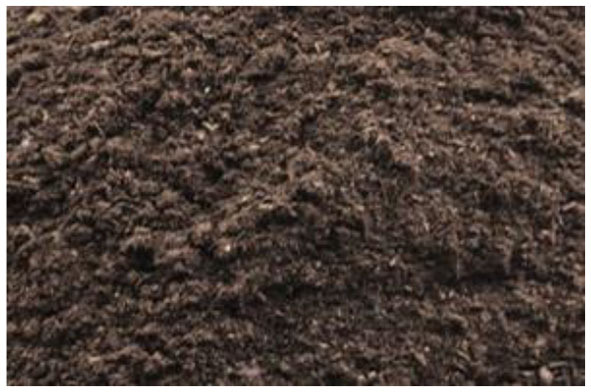
Dried olive wastewater obtained by means of a spray drier system.

The drying process of OMWW (Figures 1, 2) was achieved through a system designed and built for aroma and extract treatments, therefore, in accordance with Legislative Decree no. 17/2010 (46), is considered a food machine. Spray drying involves atomizing a liquid into very small droplets within a hot drying gas which leads to rapid drying of the droplets into solid particles. In particular, before performing the drying procedure, OMWW was treated with maize maltodextrin (40–60%) to obtain a 30% dry residue. Using a peristaltic pump, placed under the door of an accumulation tank, the treated olive mill wastewater, at an initial temperature of approximately 20°C, is continuously and regularly carried to the atomizer, which distributes it in the form of a spray inside a tank, the drying chamber, where hot and dry air flows. The stream of hot air, suitably filtered from a methane heater and sucked by an electric fan through a head with adjustable vanes, makes the water evaporate instantly and consequently lower the temperature of the mixture. The droplets heat up until evaporation begins with a consequent decrease in the size of the drops up to obtain only the dried product at the desired degree of humidity (Figure 3).

The evaporation of moisture from the drops and the formation of dry granules takes place under controlled conditions of temperature (100°C) and airflow. The moisture of the final product was 7%.

From the data obtained (Table 1), DOMWW registered content of oleuropein of 103 mg/kg and resulted richer in its derivatives (6,556 mg/kg dry weight). Moreover, ligstroside derivatives were 279 mg/kg dry weight. These compounds are very important for their blood pressure-lowering effects and health benefits and for their anti-inflammatory and antioxidant properties for fighting atherosclerosis, diabetes, cancer, neurodegenerative diseases, and even arthritis (45, 46).

**Table 1:** Phenols, expressed as mg/kg in dried olive mill wastewater (DOMWW) obtained by liquid chromatography–tandem mass spectrometry.

**Phenolic compound**	**mg/kg dry weight**
Catecol	6,12 ± 2,03
Tyrosol	2,043 ± 309
Vanillin	27,70 ± 2,26
Hydroxytyrosol	1,481± 106
Hydroxytyryloleate	564 ± 79
p-Cumaric acid	5,01 ± 1,41
Caffeic acid	2,89 ± 0,49
Apigenin	9,55 ± 0,31
Luteolin	62,38 ± 2,78
Diosmetin	3,58 ± 0,60
Luteolin-7-*O*-glucoside	88,55 ± 10,82
Luteolin-4-*O*-glucoside	11,48 ± 0,92
Oleuropein	103 ± 7
Oleuropein derivatives	6,556 ± 277
Ligstroside derivatives	279 ± 38
Rutin	48,52 ± 4,11
Verbascoside	700 ± 140
Sum of phenols	11,986 ± 437

*The data represent the mean values of tree replications with their relative SD (RSD)*.

The number of substituted phenols in DOMWW were as follows: hydroxytyrosol (1,481 mg/kg), tyrosol (2,043 mg/kg dry weight), catecol (6,12 mg/kg dry weight), vanillin (27,70 mg/kg dry weight), *p*-cumaric acid (5,01 mg/kg dry weight), caffeic acid (2,89 mg/kg dry weight), and hydroxytyrosyl oleate (564 mg/kg dry weight). All of them are powerful antioxidants and have antitumor, antimicrobial, antivirus, and anti-inflammatory properties. Among them, hydroxytyrosol and tyrosol are the ones with the most marked biological activity (47).

Interestingly, hydroxytyrosyl oleate, not present in intact olives, was detected in DOMWW representing a significant form in which hydroxytyrosol occurs. Lately, several studies have been focusing on the synthesis and evaluation of several hydroxytyrosyl fatty esters characterized by different acyl chains for their enhanced antioxidant activity compared with Hytyr. In fact, Hytyr shows low bioavailability, with fast absorption and elimination in humans which limits its use as a dietary supplement as well as an additive in foods (48). On the contrary, HtyOle has been proved to inhibit the expression of inducible NO synthase, cyclooxygenase-2, and interleukin-1β. Moreover, HtyOle revealed a significant and concentration-dependent suppression of prostaglandin E2 production (32, 51).

The flavonoids quantitated in DOMWW were apigenin (9,55 mg/kg dry weight), luteolin (62,38 mg/kg dry weight), luteolin-7-*O*-glucoside (88,55 mg/kg dry weight), luteolin-4-*O*-glucoside (11,48 mg/kg dry weight), diosmetin (3,58 mg/kg dry weight), rutin (48,52 mg/kg dry weight), and verbascoside (700 mg/kg dry weight) [(49)]. Lutein and relative glucosides (-4 and -7-*O*-glucosides), having multiple biological properties, such as anti-inflammatory, anti-allergic, and anti-tumor, work biochemically as antioxidants than as pro-oxidants. The biological effects of these compounds could be functionally related to each other, for example, the anti-inflammatory activity can be linked to its antitumor property. The anticancer properties of these substances are associated with the induction of apoptosis and the inhibition of cell proliferation, metastasis, and angiogenesis.

Luteolin-7-*O*-glucoside and luteolin-4-*O*-glucoside are, also, responsible for the color of the drupes (49–51). Rutin and verbascoside are powerful antioxidants: rutins have shown vasoprotector abilities and can strengthen the capillary wall while verbascosides possess antineoplastic properties in addition to numerous wound-healing and neuroprotective properties. Due to these properties, they are used in the dermo-cosmetological field in the topical therapy of capillary fragility and to relieve peripheral circulation disorders (52).

Apigenin and diosmetin have been the object of intense studies in the last few decades for their different beneficial effects on human health. Apigenin's activity is mainly linked to its antioxidant effect, its capacity to decrease proliferative growth, the arrest of the cell cycle, and its proapoptotic properties (53–56).

Diosmetin's activity was assayed on different types of cancer cells such as breast cancer, hepatocellular carcinoma, melanoma, colon cancer, acute myeloid leukemia, prostate cancer, NSLC, and radioresistant lung cancer (57–59). The results discussed above clearly demonstrate that drying OMWW using a spray drier set at low temperatures and after 1 month of their storage still produced an excellent extract rich in phenols with different biological properties.

## Conclusions

The three-phase olive oil extraction process produces large quantities of wastewater, which is difficult to dispose of. Vegetation water is highly toxic and acidic for plants, and to be used in agricultural practices, certain agricultural and legislative issues must be considered. The shedding on agricultural land of vegetation waters has complications due to the presence of phenols that inhibit the activity of enzymes and microorganisms responsible for anaerobic degradation of the same waters.

However, it should not be underestimated that olive mill wastewaters are rich sources of bioactive substances with various biological properties that can be used as ingredients in the food industry for obtaining functional and nutraceutical foods as well as in the pharmaceutical industry. By using a spray drier system at low temperatures, an excellent extract rich in phenols with different biological properties was produced.

## Data Availability Statement

The raw data supporting the conclusions of this article will be made available by the authors, without undue reservation.

## Author Contributions

EP, SC, and CF: Conceptualization. EP, CB, ER, and MP: Methodology. EP, CB, CF, and MP: Formal analysis. CB and ER: Data curation. EP, CB, and ER: Writing—original draft preparation, writing, reviewing, and editing. EP and SC: Supervision and project administration. All authors have read and agreed to the published version of the manuscript.

## Funding

This work was supported by the Italian Ministry of Economic Development under Horizon 2020 PON I&C 2014-2020 Project: Produzione di mangimi a valenza nutraceutica attraverso l'uso di sottoprodotti dell'industria olearia con studio degli effetti sul benessere animale e la qualità funzionale di latte e formaggi (SANSINUTRIFEED) and under the Project: Innovazioni tecnologiche nella filiera dell'oliva da olio e da mensa (INNOLITEC) (D.M. 37067/7110/2018).

## Conflict of Interest

CF was employed by company Olearia S.O.D. The remaining authors declare that the research was conducted in the absence of any commercial or financial relationships that could be construed as a potential conflict of interest.

## Publisher's Note

All claims expressed in this article are solely those of the authors and do not necessarily represent those of their affiliated organizations, or those of the publisher, the editors and the reviewers. Any product that may be evaluated in this article, or claim that may be made by its manufacturer, is not guaranteed or endorsed by the publisher.
